# Acidosis and acute kidney injury in severe malaria

**DOI:** 10.1186/s12936-018-2274-9

**Published:** 2018-03-23

**Authors:** Natthida Sriboonvorakul, Aniruddha Ghose, M. Mahtab Uddin Hassan, Md. Amir Hossain, M. Abul Faiz, Sasithon Pukrittayakamee, Kesinee Chotivanich, Yaowalark Sukthana, Stije J. Leopold, Katherine Plewes, Nicholas P. J. Day, Nicholas J. White, Joel Tarning, Arjen M. Dondorp

**Affiliations:** 1 Department of Clinical Tropical Medicine, Faculty of Tropical Medicine, Mahidol University, Bangkok, Thailand; 2 Department of Protozoology, Faculty of Tropical Medicine, Mahidol University, Bangkok, Thailand; 3 Mahidol-Oxford Tropical Medicine Research Unit, Faculty of Tropical Medicine, Mahidol University, 420/6 Rajvithi Road, Bangkok, 10400 Thailand; 4 Centre for Tropical Medicine, Nuffield Department of Clinical Medicine, University of Oxford, Oxford, UK; 5 Chittagong Medical College Hospital, Chittagong, Bangladesh

**Keywords:** Severe malaria, Acidosis, Acute kidney injury

## Abstract

**Background:**

In severe falciparum malaria metabolic acidosis and acute kidney injury (AKI) are independent predictors of a fatal outcome in all age groups. The relationship between plasma acids, urine acids and renal function was investigated in adult patients with acute falciparum malaria.

**Methods:**

Plasma and urinary acids which previously showed increased concentrations in proportion to disease severity in patients with severe falciparum malaria were quantified. Patients with uncomplicated malaria, sepsis and healthy volunteers served as comparator groups. Multiple regression and multivariate analysis were used to assess the relationship between organic acid concentrations and clinical syndromes, in particular AKI.

**Results:**

Patients with severe malaria (n = 90), uncomplicated malaria (n = 94), non-malaria sepsis (n = 19), and healthy volunteers (n = 61) were included. Univariate analysis showed that both plasma and creatinine-adjusted urine concentrations of *p*-hydroxyphenyllactic acid (pHPLA) were higher in severe malaria patients with AKI (*p* < 0.001). Multiple regression analysis, including plasma or creatinine-adjusted urinary acids, and PfHRP2 as parasite biomass marker as independent variables, showed that pHPLA was independently associated with plasma creatinine (β = 0.827) and urine creatinine (β = 0.226). Principal component analysis, including four plasma acids and seven urinary acids separated a group of patients with AKI, which was mainly driven by pHPLA concentrations.

**Conclusions:**

Both plasma and urine concentrations of pHPLA closely correlate with AKI in patients with severe falciparum malaria. Further studies will need to assess the potential nephrotoxic properties of pHPLA.

**Electronic supplementary material:**

The online version of this article (10.1186/s12936-018-2274-9) contains supplementary material, which is available to authorized users.

## Background

Severe malaria caused by *Plasmodium falciparum* remains a major cause of death in the tropics, with an estimated 429,000 deaths in 2015 [1]. The major manifestations of severe malaria, which independently predict fatal outcome in both adults and children, include coma (cerebral malaria), acidosis and renal dysfunction [2–5]. Coma in cerebral malaria is characterized by an absence of localizing neurological signs, and full recovery in most adults and the majority of children if patients survive. Acidosis in severe malaria is primarily a lactic acidosis, resulting from anaerobic glycolysis as a result of microcirculatory impairment caused by sequestered parasitized red cells. This is compounded by reduced hepatic lactate clearance [6]. But lactic acid comprises less than half of the acid load, whereas these incompletely characterized additional acids are an independent prognosticator for a fatal outcome [7]. Acute kidney injury (AKI) is common in adult severe malaria, and may be under-recognized in paediatric patients [8, 9]. The pathology of AKI is characterized by acute tubular injury, associated with an accumulation of parasitized red cells and mononuclear cells in the glomerular and peritubular capillaries with a contribution of haem-mediated oxidative damage [10–12]. Metabolic acidosis and AKI are interrelated, since renal dysfunction causes accumulation of acids normally excreted or metabolized by the kidney. Indeed, in severe malaria plasma creatinine is an independent predictor of acidosis [6]. The plasma concentration of an acid, or other biomarkers of severity may therefore reflect either increased production, reduced clearance or both. Adjusting the plasma concentration for plasma creatinine only corrects for concomitant renal dysfunction if the acid or biomarker has similar renal clearance dynamics as creatinine, including similar renal filtration, re-absorption and secretion. For many biomarkers these properties have not been assessed.

The current study reports a secondary data analysis on plasma and urine organic acids elevated in adult patients with falciparum malaria and sepsis [13, 14], and relates these to organ failure in severe malaria, in particular AKI.

## Methods

### Patients and sample collection

Details of the study have been described previously in detail [13, 14]. Written informed consent was obtained from all participants in the study or from their attending relative for incapacitated patients. Consecutive adult patients with slide-proven severe falciparum malaria admitted at Chittagong Medical College Hospital, Chittagong, Bangladesh, were recruited in the study. Pregnant women were excluded from the study. In addition, a sub-set of Bangladeshi patients with other sepsis syndromes with acidosis and with uncomplicated falciparum malaria were included in the study. In addition, a group of healthy controls were recruited from the local population, screened by a physical exam and basic laboratory testing.

Severe malaria was classified by modified World Health Organization (WHO) criteria [15]. AKI was defined by a plasma creatinine > 265 μmol/l, coma as a Glasgow Coma Score (GCS) < 11 and a high parasite biomass by a plasma concentration of *P. falciparum* histidine-rich protein 2 (*Pf*HRP2) > 1000 ng/ml [16].

Non-malaria sepsis was defined by a blood film negative for malaria of all species, clinical suspicion of bacterial infection, based on history, examination and, when available, complete blood count and other investigations, a standard base deficit (SBD) ≥ 3 mEq/l and 2 or more of the following four criteria: (1) heart rate > 90 beats per minute; (2) respiratory rate > 20 breaths per minute; (3) systolic blood pressure < 90 mmHg; and/or, (4) GCS score < 15. Recruitment in this study was prior to the recently adopted updated criteria for sepsis [17].

Healthy volunteers were screened by microscopy for absence of *Plasmodium* parasites. Ethical approval for this observational study was provided by the Chittagong Medical College Hospital Ethical Review Committee and the Oxford Tropical Research Ethics Committee.

### Analytical methods

The analytical method for quantifying organic acids in human plasma and urine have been described previously [13]. Selection of acids was based on their increased plasma and urine concentrations observed in a pilot study and included l-lactic acid (LA), α-hydroxybutyric acid (αHBA), β-hydroxybutyric acid (βHBA) and *p*-hydroxyphenyllactic acid (pHPLA). In addition, methylmalonic (MMA), ethylmalonic (EMA) and α-ketoglutaric acids (αKGA) were quantified only in urine, because concentrations in plasma were lower than the limit of detection [13]. Samples were purified initially by high-throughput strong anion exchange solid-phase extraction in a 96-well plate format. Reconstituted extracts were separated, detected and quantified by hydrophilic interaction liquid chromatography (HILC) coupled to negative mass spectrometry (MS). The LC–MS method was validated according to US Food and Drug Administration guidelines [13]. Measured urine concentrations were corrected for renal function by adjusting for urine and plasma creatinine concentrations, as described previously [18]

$$\left[ A \right]_{urine - corrected} = \left[ A \right]_{urine} \times \frac{{\left[ {Creatinine} \right]_{plasma} }}{{\left[ {Creatinine} \right]_{urine} }}.$$

Peripheral blood parasitaemia was assessed by microscopy from peripheral blood slides stained according to Field’s method. Plasma *Pf*HRP2 concentration was assessed by a commercial sandwich enzyme-linked immunosorbent assay kit (ELISA) (Cell Labs, Brookvale, Australia), as described previously [19].

Plasma *Pf*HRP2, released by the parasitized red blood cell at schizont rupture, is a surrogate measure of the total body parasite biomass, which includes the sequestered biomass [19].

### Statistical methods

Plasma and corrected urine acid concentrations were log-normalized. Acid concentrations were compared initially between disease groups and disease manifestations. The four groups (severe malaria, uncomplicated malaria, non-malaria sepsis, healthy) were compared by analysis of variance (ANOVA). Student’s t test was used where only two participant groups were compared. Multiple regression models were assessed for plasma and urinary acids, *Pf*HRP2 as independent variables, and plasma or urinary creatinine as dependent variable. For the multiple regression analysis, acid concentrations were log-transformed toward normality before analysis. All variables in univariate analysis were initially included in the model then backward stepwise selection was performed, removing variables with α ≥ 0.05 and using optimal value of Akaike’s information criterion (AIC) and Bayesian information criterion (BIC) in the model.

Further multivariate statistical analyses relating acid concentrations to disease states included principal component analysis (PCA) and partial least square discrimination analysis (PLSDA). PCA analysis was used to visualize clustering for specific disease profiles, including presence of AKI, coma and parasite biomass. PLSDA analysis was then used to create prediction models to classify unknown samples.

Analyses were performed with STATA/IC 12.0 software (StataCorp, College Station, TX, USA), MATLAB version 7.11 software (MathWorks, Natick, MA, USA) and GraphPad PRISM^®^ version 5.03 (GraphPad Software Inc, CA, USA).

## Results

### Patient characteristics and univariate analysis

Baseline clinical and laboratory characteristics are summarized in Table 1. The concentrations of plasma and corrected urinary LA, αHBA, βHBA, and pHPLA were higher in patients with severe malaria compared to uncomplicated malaria. Creatinine-corrected urinary concentrations of LA, αHBA, βHBA, pHPLA, MMA, EMA and αKGA were also higher in patients with severe compared to uncomplicated malaria.

**Table 1:** Baseline clinical and laboratory

Characteristics	Severe malaria N = 90	Uncomplicated malaria N = 94	Sepsis N = 19	Healthy N = 61^d^	*p* value
Male, n (%)	63 (70%)	74 (79%)	9 (47%)		0.009
Age (year)*	32 (25–45)	29 (21–45)	35 (25–48)		0.205
Glasgow Coma Scale score*	10 (8–14)	15 (15–15)	14 (8–15)		< 0.001
GCS score < 11, n (%)	59/90 (66%)	0 (0%)	6/19 (32%)		
Parasitaemia (count/μl)	41,929 (26,191–67,127)	10,287 (5959–17,758)			< 0.001
PfHRP2 (ng/ml)	1740 (1333–2272)	240.3 (167.8–344.1)			< 0.001
Total bilirubin (mg/dl)	2.8 (2.3–3.5, 0.2–44.2)	1.1 (0.9–1.4, 0.2–28.3)	0.94 (0.59–1.49)		< 0.001
Creatinine (mg/dl)	1.48 (1.34–1.65)	0.95 (0.89–1.01)	1.68 (1.25–2.26)		< 0.001
Acute kidney injury (creatinine > 3 mg/dl), n (%)	13/90 (14%)	0/94 (0%)	4/19 (21%)		
Plasma concentration (μmol/l)^a^					
LA	4587 (4028–5223)	2246 (2080–2426)	2849 (2148–3779)	1111 (1088–1133)	< 0.001
aHBA	118.7 (106–133)	63.07 (56.58–70.3)	107.7 (86.2–134.6)	22.18 (21.19–23.21)	< 0.001
bHBA	139.0 (126.1–153.1)	101.9 (91.28–113.7)	247.9 (160.4–383.2)	71.37 (69.43–73.37)	< 0.001
pHPLA	9.73 (8.51–11.11)	5.85 (5.57–6.15)	8.24 (6.97–9.73)	1.06 (0.99–1.14)	< 0.001
Corrected urine concentrations (μmol/mmol CrCl)^a, b^					
LA	9.94 (7.54–13.09)	2.85 (2.32–3.49)	13.82 (4.76–40.1)		< 0.001
aHBA	0.95 (0.73–1.25)	0.63 (0.51–0.78)	1.07 (0.55–2.09)		0.063
bHBA	2.56 (2.17–3.03)	1.88 (1.57–2.26)	4.61 (2.13–9.96)		0.003
pHPLA	1.42 (1.09–1.86)	0.48 (0.34–0.66)	0.83 (0.30–2.29)		< 0.001
MMA	0.14 (0.12–0.17)	0.23 (0.19–0.28)	0.15 (0.11–0.22)		< 0.001
EMA	0.21 (0.17–0.25)	0.34 (0.27–0.43)	0.32 (0.21–0.48)		0.005
aKGA	2.44 (2.09–2.85)	2.16 (1.89–2.47)	4.31 (2.77–6.70)		< 0.001
Urine concentrations (μmol/l)^c^					
LA	560.0 (436.3–718.7)	166.8 (140.0–198.7)	370.7 (196.7–698.7)	247.6 (221.1–277.2)	< 0.001
aHBA	56.58 (44.39–72.12)	35.97 (29.98–43.16)	35.44 (23.43–53.59)	90.71 (80.0–102.8)	< 0.001
bHBA	159.4 (142.2–178.7))	127.0 (114.4–140.9)	252.3 (168.7–377.3)	122.1 (115.3–129.2)	0.007
pHPLA	74.98 (60.85–92.40)	26.31 (20.73–33.40)	28.57 (17.75–45.97)	21.76 (16.29–29.08)	< 0.001
MMA	10.19 (8.51–12.20)	13.32 (11.20–15.84)	6.99 (4.53–10.79)	24.25 (20.79–28.28)	< 0.001
EMA	13.22 (10.63–16.45)	19.70 (15.83–24.52)	11.79 (7.51–18.53)	45.34 (36.54–56.26)	< 0.001
aKGA	218.0 (201.5–235.9)	220.8 (201.9–241.4)	250.1 (190.8–327.8)	UD	< 0.001

Values are geometric mean (95% CI), except * median (IQR)

^a^For all plasma specimens, EMA, MMA, α-KGA were assayed but undetectable

^b^Urine creatinine-adjusted concentrations were corrected as described in the “Methods” section

^c^Actual urine concentrations

^d^For healthy participants, clinical parameters and creatinine values were not available

Plasma and urine acid concentrations in patients with severe malaria according to the presence of AKI are summarized in Table 2. In the univariate analysis, plasma concentrations of pHPLA and βHBA were higher in patients with AKI. Of these acids, only pHPLA concentrations were also increased in the urine of patients with AKI. When adjusted for urine creatinine concentrations, the urine concentrations of LA, βHBA, pHPLA, and αKGA were all increased in patients with AKI. In contrast, urine concentrations of αHBA, MMA and EMA were lower in patients with AKI. Univariate analysis of acid profiles according to presence of coma (as GCS<11) or high parasite biomass (reflected by elevated plasma *Pf*HRP2 concentrations) did not show differences in acid concentrations in patients with or without either syndrome (Additional file 1: Table S1a and Table S1b).

**Table 2:** Plasma and urine concentrations of organic acids detected in patients with severe malaria with or without acute kidney injury (AKI)

Characteristics	Severe malaria	Severe malaria	p value
	with AKI (N = 13)	without AKI (N = 77)	
Plasma concentration (μmol/l)^a^			
LA	6406 (4816–8520)	4335 (3760–4998)	0.066
αHBA	142 (107–188)	115 (102–131)	0.348
βHBA	178 (133–238)	133 (120–148)	0.043
pHPLA	33.8 (22.5–50.7)	7.88 (7.36–8.45)	< 0.001
Urine creatinine adjusted concentrations (μmol/mmol CrCl)^a, b^			
LA	50.7 (30.4–84.5)	7.55 (5.78–9.86)	< 0.001
αHBA	1.33 (0.86–2.06)	0.90 (0.66–1.22)	0.577
βHBA	5.94 (4.52–7.80)	2.22 (1.87–2.64)	< 0.001
pHPLA	8.80 (6.17–12.5)	1.04 (0.82–1.33)	< 0.001
MMA	0.11 (0.07–0.17)	0.15 (0.12–0.18)	0.245
EMA	0.16 (0.09–0.25)	0.22 (0.18–0.27)	0.232
αKGA	7.29 (6.28–8.46)	2.03 (1.76–2.33)	< 0.001
LA	800 (451–1420)	527 (400–696)	0.871
αHBA	21.0 (13.7–32.3)	67.0 (51.8–86.8)	0.019
βHBA	101 (86.0–120)	172 (152–194)	0.012
pHPLA	139 (102–189)	67.6 (53.6–85.2)	0.019
MMA	3.21 (2.59–3.98)	11.2 (9.38–13.4)	0.032
EMA	3.58 (2.42–5.30)	15.4 (12.4–19.1)	0.049
αKGA	242 (213–275)	218 (201–237)	0.197

For plasma specimens, undetectable concentrations were treated as half the lower limit of detection to allow inclusion in intergroup comparisons. For creatinine-corrected urine concentrations, undetectable values were treated as half the lowest value calculated from a detectable specimen. Values are geometric mean (95% CI)

GCS, Glasgow Coma Scale; geo mean, geometric mean; Hb, haemoglobin; PfHRP2, *Plasmodium falciparum* histidine-rich protein 2; UD, undetectable; LA, lactic acid; HPLA, hydroxyphenyllactic acid; α-HBA, α-hydroxybutyric acid; β-HBA, β-hydroxybutyric acid; EMA, ethylmalonic acid; MMA, methylmalonic acid; α-KGA, α-ketoglutaric acid; CrCl. creatinine clearance

^a^For all plasma specimens, EMA, MMA, α-KGA were assayed but undetectable

^b^Urine creatinine-adjusted concentrations were corrected as described in the “Methods” section

The (log) plasma concentrations of pHPLA correlated with (log) plasma concentrations of *Pf*HRP2, (r = 0.57, p < 0.001). The (log) corrected urine concentration of pHPLA also correlated with plasma PfHRP2 (r = 0.38, p < 0.001), whereas the unadjusted pHPLA urine concentrations did not (r = -0.024, p = 0.835). The (log) plasma concentrations of pHPLA also correlated with the (log) plasma total bilirubin concentrations (r = 0.46, p < 0.001).

### Multiple regression and multivariate analysis

In patients with severe malaria, applying linear multiple regression models with (log) plasma creatinine as dependent variable, including plasma acids (LA, αHBA, βHBA, pHPLA) and plasma *Pf*HRP2 as marker for total parasite burden as independent variables, plasma pHPLA and LA were retained in the model (Additional file 1: Table S2). In the model, pHPLA was associated most strongly with plasma creatinine (β = 0.827).

In patients with sepsis, linear regression models with (log) plasma creatinine as dependent variable, including plasma acids (LA, αHBA, βHBA, pHPLA) as independent variables (Additional file 1: Table S3), only the (log) plasma pHPLA concentration was correlated with the (log) plasma creatinine (r = 0.26, p = 0.007) and plasma pHPLA was associated most strongly with plasma creatinine, with β = 0.849, Plasma LA, αHBA and βHBA did not show a correlation.

**Fig. 1:**
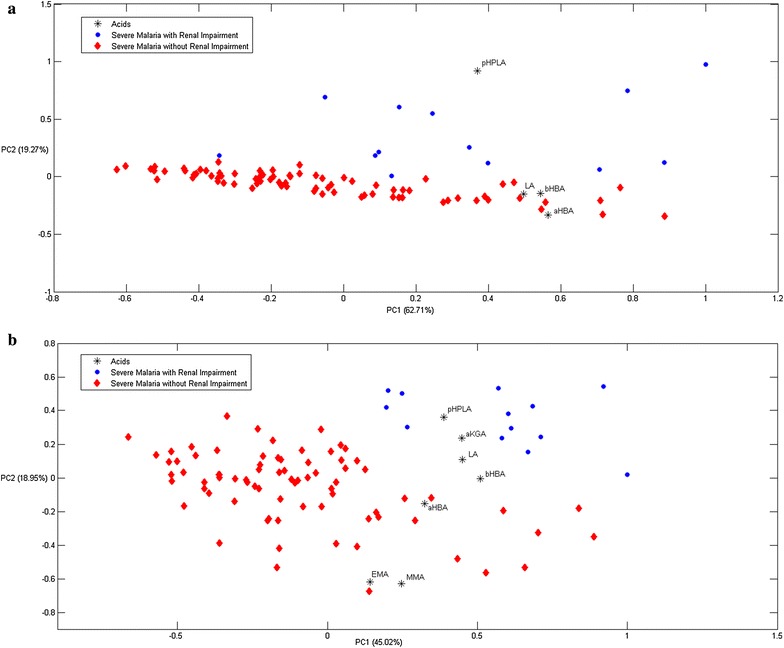
**a** Principal component analysis (PCA) results of plasma concentrations of l-lactic acid (LA), α-hydroxybutyric acid (αHBA), β-hydroxybutyric acid (βHBA), and *p*-hydroxyphenyllactic acid (HPLA). The second component (PC2) separates out a group of patients with renal impairment (in blue), which is largely driven by the pHPLA plasma concentration (factor loading HPLA = 0.92). **b** Principal component analysis (PCA) results of corrected urine concentrations of l-lactic acid (LA), α-hydroxybutyric acid (αHBA), β-hydroxybutyric acid (βHBA) and p-hydroxyphenyllactic acid (HPLA), methylmalonic acid (MMA), ethylmalonic acid (EMA) and α-ketoglutaric acid (αKGA). The second component (PC2) separates out a group of patients with renal impairment (in blue)

Principal component analysis analysis was performed to assess clustering of acids with certain disease manifestations (presence of AKI, coma and high parasite biomass) (Fig. 1 and Additional file 1: Figures S3–S6). In patients with AKI, clear clustering could be observed. PCA analysis including all four acids assessed in plasma separated a group of patients with AKI along the PC2 axis (Fig. 1a). Related factor loading results for the PC2 component were: LA = − 0.15, αHBA = − 0.33, βHBA = − 0.15, and pHPLA = 0.92, which shows that the separation was mainly driven by the HPLA plasma concentrations. When plasma creatinine was included in the PCA analysis, a similar separation was apparent (Additional file 1: Figure S1), with factor loading results for the PC2 component of − 0.65 for creatinine and − 0.51 for pHPLA. PCA analysis including urine concentration of seven acids (corrected for creatinine) also showed separation of patients with AKI along the PC2 axis (Fig. 1b). The related factor loading results for the PC2 component were: LA = 0.11, αHBA = − 0.15, βHBA = 0.00, HPLA = 0.36, MMA = − 0.63, EMA = − 0.62, and αKGA = 0.24, showing that pHPLA together with LA, βHBA and αKGA were the main drivers of the separation. When urinary creatinine was included in the PCA analysis, a similar separation was apparent (Additional file 1: Figure S2). PLSDA including plasma and urine acid concentrations, showed a high recognition ability for AKI of 92.3% for plasma acids and 99.4% for urinary acids. The leave-one-out cross validation method (LOOCV) showed similar high recognition ability (Additional file 1: Tables S4a, b).

## Discussion

In patients with severe falciparum malaria the concentrations of several small organic acids are increased compared to patients with uncomplicated malaria and healthy controls. Apart from lactic acid, concentrations of pHPLA, αHBA and βHBA in plasma and pHPLA, αHBA, βHBA, MMA, EMA, and αKGA in urine are all elevated [14]. In this secondary analysis the relationships between these acid concentrations and specific organ involvement, in particular AKI, were assessed in more detail.

Increased concentrations of plasma LA, βHBA and pHPLA were observed in severe malaria patients with AKI compared to patients without AKI. The main source of plasma lactate in severe malaria is from anaerobic glycolysis in the human host caused by blockage of the microcirculation by sequestered parasitized red blood cells and compounded by reduced hepatic clearance [6, 7, 20]. *Plasmodium falciparum* parasites also produce lactate, but this is thought to be a minor contribution to the total lactate concentration [7]. Plasma lactate and AKI both have strong prognostic significance for death in severe malaria.

Urinary lactate concentrations were also increased in severe malaria patients with AKI. In healthy subjects, creatinine has limited tubular re-absorption and negligible tubular secretion, whereas lactate is fully filtered by the glomerulus [21] and then almost completely re-absorbed in the proximal tubule [21]. Tubular re-absorption of lactate might be disturbed in severe malaria patients with AKI, characterized by acute tubular injury. Increased renal production of lactate is another possible contributor, related to the extensive sequestration of parasitized red blood cells in renal vessels, shown in *post*-*mortem* autopsy studies of severe malaria-related AKI [11], whereas the medulla of the kidney is already scarcely vascularized.

βHBA is a ketoacid generated from human free fatty acid metabolism, which becomes an energy source during fasting, dysregulated type I diabetes, and other disease states. Patients with severe malaria have usually been fasting for an extended period, and there is a high metabolic demand related to the severe febrile illness. As previously shown, plasma concentrations of βHBA are elevated in severe falciparum malaria, provided ketogenesis has not been suppressed by quinine-induced insulin secretion, and correlate well with plasma creatinine concentrations (r = 0.68, p = 0.001) [14, 22–24].

Ketogenesis can also occur in the kidney, which might contribute to the increased urine concentrations of βHBA adjusted for plasma and urinary creatinine concentrations.

In the present study, an increase in both plasma and urinary concentrations of pHPLA in patients with severe malaria was shown, strongly correlating with the presence of AKI. This indicates that pHPLA production is increased in these patients, although reduced hepatic clearance could also have contributed. The renal handling of pHPLA has not been well characterized, so it cannot be excluded that the increased urine concentrations are explained by reduced renal tubular re-absorption of glomerular-filtered pHPLA. Local renal production of pHPLA is an alternative explanation. pHPLA is derived from *p*-hydroxyphenylpyruvic (pHPPA) acid and a product in the phenylalanine and tyrosine pathways. Plasma phenylalanine (an indirect precursor of pHPPA) has shown to be increased in adult patients with severe malaria [25]. Both the human host and *P. falciparum* produce pHPLA (Human Metabolome Database (HMDB) [26]. The human gut flora produces the d-isomer of pHPLA, and is thus another potential source. The LC–MS method used in the current study cannot distinguish between d and l-form isomers. Plasma *Pf*HRP2 and pHPLA concentrations were correlated and might suggest *P. falciparum* as a potential source of pHPLA in patients with severe malaria. However, a positive correlation with *Pf*HRP2 may also reflect increased sequestration in the gut microvasculature, causing increased translocation of gut microbioma and their products.

In the PCA analysis, pHPLA was a strong driver for clustering on the PC2 axis patients with AKI, which was confirmed by PLSDA analysis. pHPLA remained a significant driver separating the patients with AKI, even when creatinine was added as a variable in the PCA analysis. The results suggest that pHPLA is not merely a biomarker for AKI, but also could contribute to renal dysfunction in patients with severe falciparum malaria. The pathogenesis of AKI in severe malaria is multifactorial. Free haemoglobin associated oxidative damage to the renal tubules is thought to be an important contributor [10]. In a large autopsy series, microvascular sequestration of parasitized red blood cells in the peritubular capillaries is observed, which could impair the already vulnerable circulation of the renal medulla. Locally parasite-produced pHPLA could be an additional contributor to renal tubular injury. In patients with tyrosinemia, pHPLA accumulates, and renal tubular damage is common in this disease [27]. In addition, pHPLA has been implicated as a cause of tubular damage in Fanconi syndrome, although alternative mechanisms have also been proposed [28]. Assessment of the sources of pHPLA in severe falciparum malaria, and its contribution to renal damage will need further investigation, including animal studies, metabolomic studies in *P. falciparum,* and detailed kinetic studies in patients, distinguishing the l- and d-isomers of pHPLA.

Limitations of the study include the focus on a limited number of acids selected by the column used for extraction. Although during the development phase several columns were compared [13], it cannot be excluded that additional contributing small organic acids have been missed. The number of sepsis patients was limited in this study, and the causing microorganisms were not identified, because of the limited local diagnostic capacity. This precludes drawing firm conclusions in this patient group. In addition, 24-h urines were not collected in the study, so that the total quantity of excreted acids could not be estimated accurately. Correcting the urine acid concentration for urine creatinine concentration partly circumvents this problem, but assumes similar renal clearance mechanisms of these compounds.

## Conclusions

This secondary analysis of organic acid concentrations in patients with falciparum malaria shows that both plasma and urine concentrations of pHPLA are independently correlated with AKI, suggesting a possible causative contribution of pHPLA to AKI. This hypothesis will now need further testing.

## Additional file

**Additional file 1: Figure S1.** Principal Component Analysis (PCA) results of plasma concentrations of L-lactic acid (LA), α-hydroxybutyric acid (αHBA), β-hydroxybutyric acid (βHBA) and p-hydroxyphenyllactic acid (pHPLA) with plasma creatinine of severe malaria patients with AKI (in blue) and without AKI(in red). **Figure S2.** Principal Component Analysis (PCA) results of corrected urine concentrations of L-lactic acid (LA), α-hydroxybutyric acid (αHBA), β-hydroxybutyric acid (βHBA), p-hydroxyphenyllactic acid (pHPLA), methylmalonic acid (MMA), ethylmalonic acid, (EMA) and α-ketoglutaric acid (αKGA) with urinary creatinine of severe malaria patients with AKI (in blue) and without AKI (in red). **Figure S3.** Principal Component Analysis (PCA) results of plasma concentrations of L-lactic acid (LA), α-hydroxybutyric acid (αHBA), β-hydroxybutyric acid (βHBA) and p-hydroxyphenyllactic acid (pHPLA) of severe malaria patients with coma (in blue) and without coma (in red). **Figure S4.** Principal Component Analysis (PCA) results of corrected urine concentrations of L-lactic acid (LA), α-hydroxybutyric acid (αHBA), β-hydroxybutyric acid (βHBA), p-hydroxyphenyllactic acid (HPLA), methylmalonic acid (MMA), ethylmalonic acid (EMA) and α-ketoglutaric acid (αKGA) of severe malaria patients with coma (in blue) and without coma (in red). **Figure S5.** Principal Component Analysis (PCA) results of plasma concentrations of L-lactic acid (LA), α-hydroxybutyric acid (αHBA), β-hydroxybutyric acid (βHBA) and p-hydroxyphenyllactic acid (HPLA) of severe malaria patients with high parasite biomass (in blue) and without high parasite biomass (in red). **Figure S6.** Principal Component Analysis (PCA) results of corrected urine concentrations of L-lactic acid (LA), α-hydroxybutyric acid (αHBA), β-hydroxybutyric acid (βHBA), p-hydroxyphenyllactic acid (HPLA), methylmalonic acid (MMA), ethylmalonic acid (EMA) and α-ketoglutaric acid (αKGA) of severe malaria patients with high parasite biomass (in blue) and without high parasite biomass (in red). **Figure S7.** Principal Component Analysis (PCA) results of uncorrected urine concentrations of L-lactic acid (LA), α-hydroxybutyric acid (αHBA), β-hydroxybutyric acid (βHBA), p-hydroxyphenyllactic acid (HPLA), methylmalonic acid (MMA), ethylmalonic acid (EMA) and α-ketoglutaric acid (αKGA) of severe malaria patients with AKI (in blue) and without AKI (in red). **Figure S8.** Principal Component Analysis (PCA) results of uncorrected urine concentrations of L-lactic acid (LA), α-hydroxybutyric acid (αHBA), β-hydroxybutyric acid (βHBA), p-hydroxyphenyllactic acid (HPLA), methylmalonic acid (MMA), ethylmalonic acid (EMA) and α-ketoglutaric acid (αKGA) with urinary creatinine of severe malaria patients with AKI (in blue) and without AKI (in red). **Table S1a.** Plasma and urine concentration range of organic acids detected in patients with severe malaria with coma (+/-). **Table S1b.** Plasma and urine concentration range of organic acids detected in patients with severe malaria with high biomass (+/-). **Table S2.** Summary of linear regression models in patients with severe falciparum malaria (N = 90), with plasma PfHRP2 concentrations and plasma or urinary acid concentrations as independent variables, and plasma or urinary creatinine concentrations as dependent variable. **Table S3.** Summary of linear regression models in patients with sepsis (N = 19), with plasma PfHRP2 concentrations and plasma or urinary acid concentrations as independent variables, and plasma or urinary creatinine concentrations as dependent. **Table S4a.** Partial Least Square Discriminant Analysis classification results of plasma concentration of 4 acids. **Table S4b.** Partial Least Square Discriminant Analysis classification results of corrected urine concentration of 7 acids.
