# Supplementary material for: Acidosis and acute kidney injury in severe malaria
Source: Malar J. 2018 Mar 23;17:128. doi: 10.1186/s12936-018-2274-9 (PMC5865348; doi:10.1186/s12936-018-2274-9)
Supplement: Supplementary file 1 — Additional file 1: Figure S1. Principal Component Analysis (PCA) results of plasma concentrations of L-lactic acid (LA), α-hydroxybutyric acid (αHBA), β-hydroxybutyric acid (βHBA) and p-hydroxyphenyllactic acid (pHPLA) with plasma creatinine of severe malaria patients with AKI (in blue) and without AKI(in red). Figure S2. Principal Component Analysis (PCA) results of corrected urine concentrations of L-lactic acid (LA), α-hydroxybutyric acid (αHBA), β-hydroxybutyric acid (βHBA), p-hydroxyphenyllactic acid (pHPLA), methylmalonic acid (MMA), ethylmalonic acid, (EMA) and α-ketoglutaric acid (αKGA) with urinary creatinine of severe malaria patients with AKI (in blue) and without AKI (in red). Figure S3. Principal Component Analysis (PCA) results of plasma concentrations of L-lactic acid (LA), α-hydroxybutyric acid (αHBA), β-hydroxybutyric acid (βHBA) and p-hydroxyphenyllactic acid (pHPLA) of severe malaria patients with coma (in blue) and without coma (in red). Figure S4. Principal Component Analysis (PCA) results of corrected urine concentrations of L-lactic acid (LA), α-hydroxybutyric acid (αHBA), β-hydroxybutyric acid (βHBA), p-hydroxyphenyllactic acid (HPLA), methylmalonic acid (MMA), ethylmalonic acid (EMA) and α-ketoglutaric acid (αKGA) of severe malaria patients with coma (in blue) and without coma (in red). Figure S5. Principal Component Analysis (PCA) results of plasma concentrations of L-lactic acid (LA), α-hydroxybutyric acid (αHBA), β-hydroxybutyric acid (βHBA) and p-hydroxyphenyllactic acid (HPLA) of severe malaria patients with high parasite biomass (in blue) and without high parasite biomass (in red). Figure S6. Principal Component Analysis (PCA) results of corrected urine concentrations of L-lactic acid (LA), α-hydroxybutyric acid (αHBA), β-hydroxybutyric acid (βHBA), p-hydroxyphenyllactic acid (HPLA), methylmalonic acid (MMA), ethylmalonic acid (EMA) and α-ketoglutaric acid (αKGA) of severe malaria patients with high parasite biomass (in blue) and witho [file 12936_2018_2274_MOESM1_ESM.docx]

**
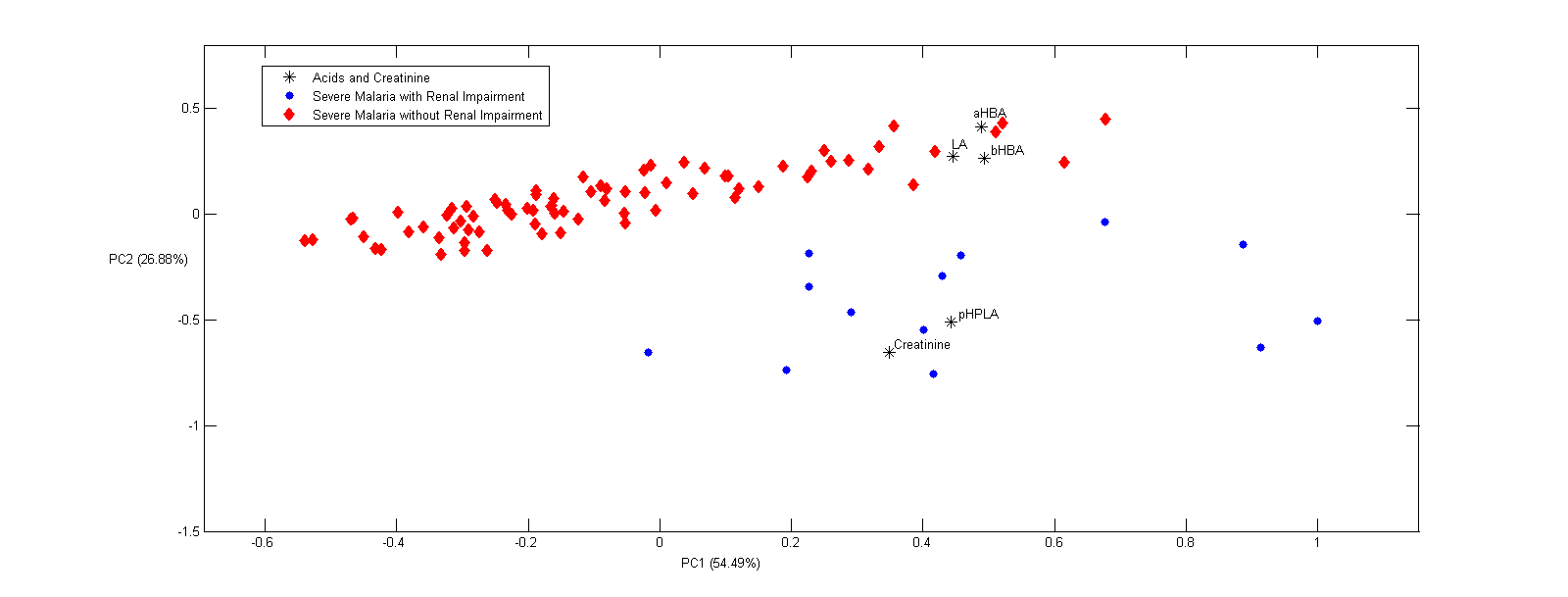
**

**Fig. S1** Principal Component Analysis (PCA) results of plasma concentrations of L-lactic acid (LA), α-hydroxybutyric acid (αHBA), β-hydroxybutyric acid (βHBA) and p-hydroxyphenyllactic acid (pHPLA) with plasma creatinine of severe malaria patients with AKI (in blue) and without AKI(in red).

**
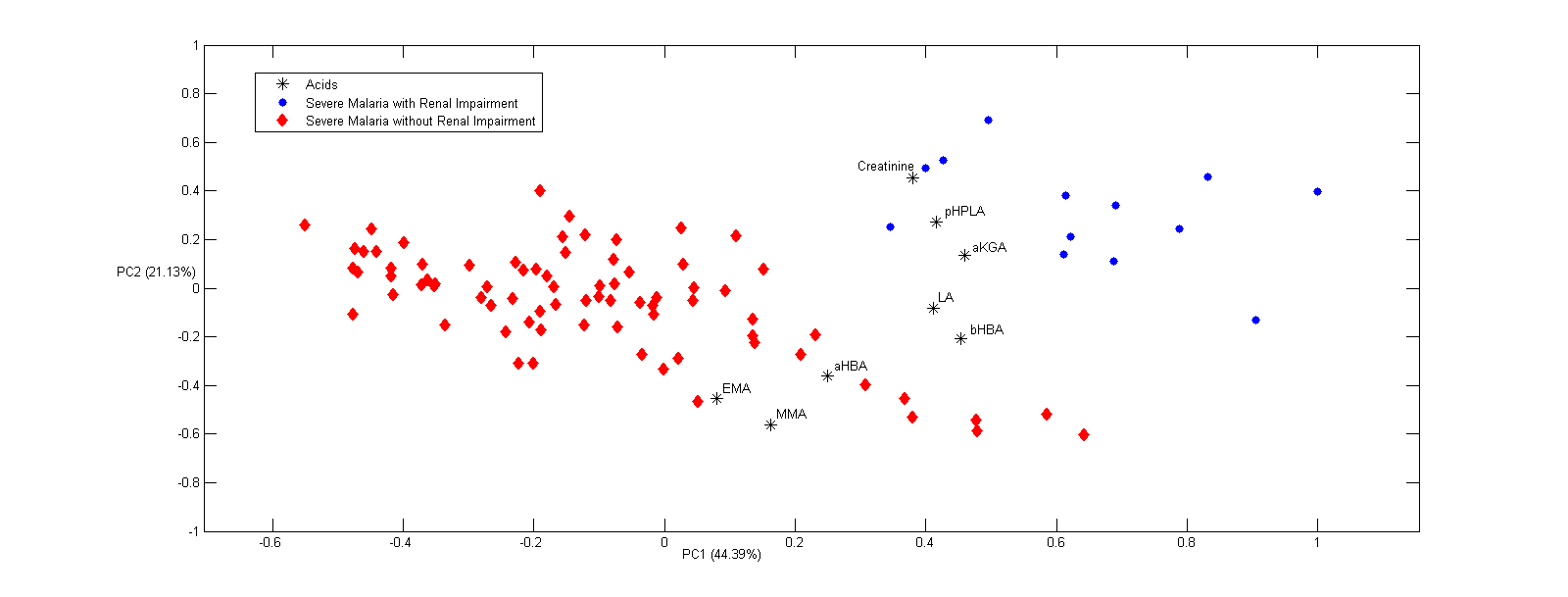
**

**Fig. S2** Principal Component Analysis (PCA) results of corrected urine concentrations of L-lactic acid (LA), α-hydroxybutyric acid (αHBA), β-hydroxybutyric acid (βHBA), p-hydroxyphenyllactic acid (pHPLA), methylmalonic acid (MMA), ethylmalonic acid, (EMA) and α-ketoglutaric acid (αKGA) with urinary creatinine of severe malaria patients with AKI (in blue) and without AKI (in red).


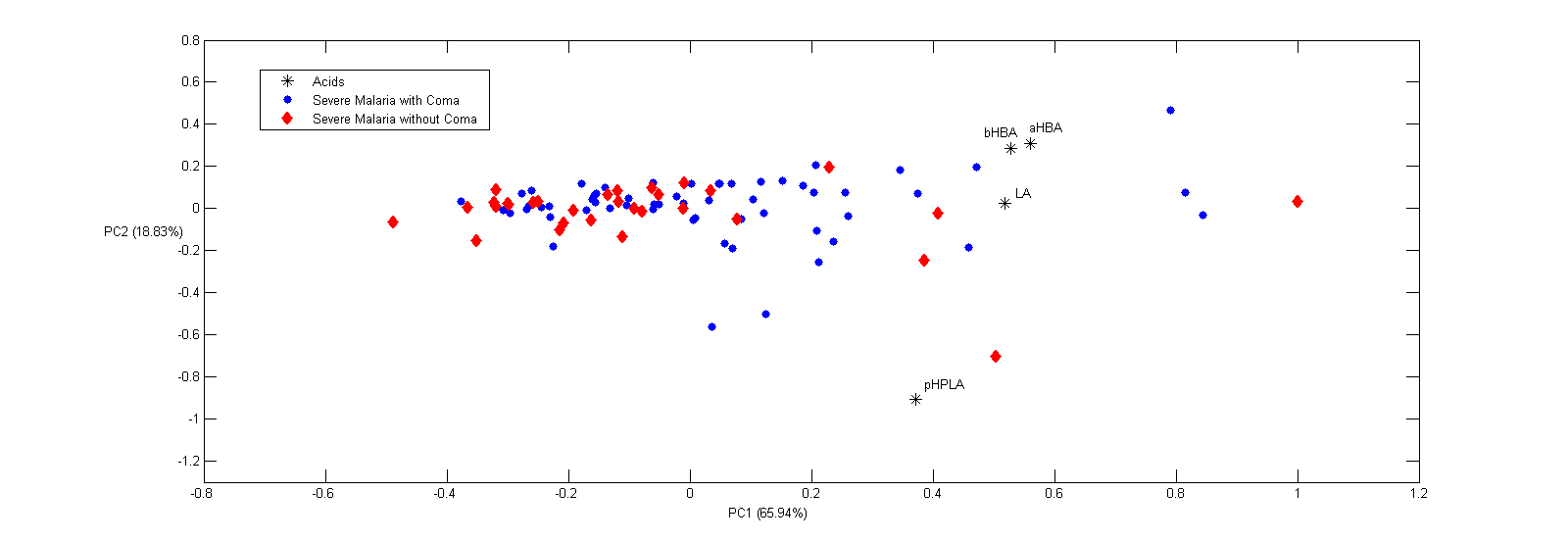


**Fig. S3** Principal Component Analysis (PCA) results of plasma concentrations of L-lactic acid (LA), α-hydroxybutyric acid (αHBA), β-hydroxybutyric acid (βHBA) and p-hydroxyphenyllactic acid (pHPLA) of severe malaria patients with coma (in blue) and without coma (in red).


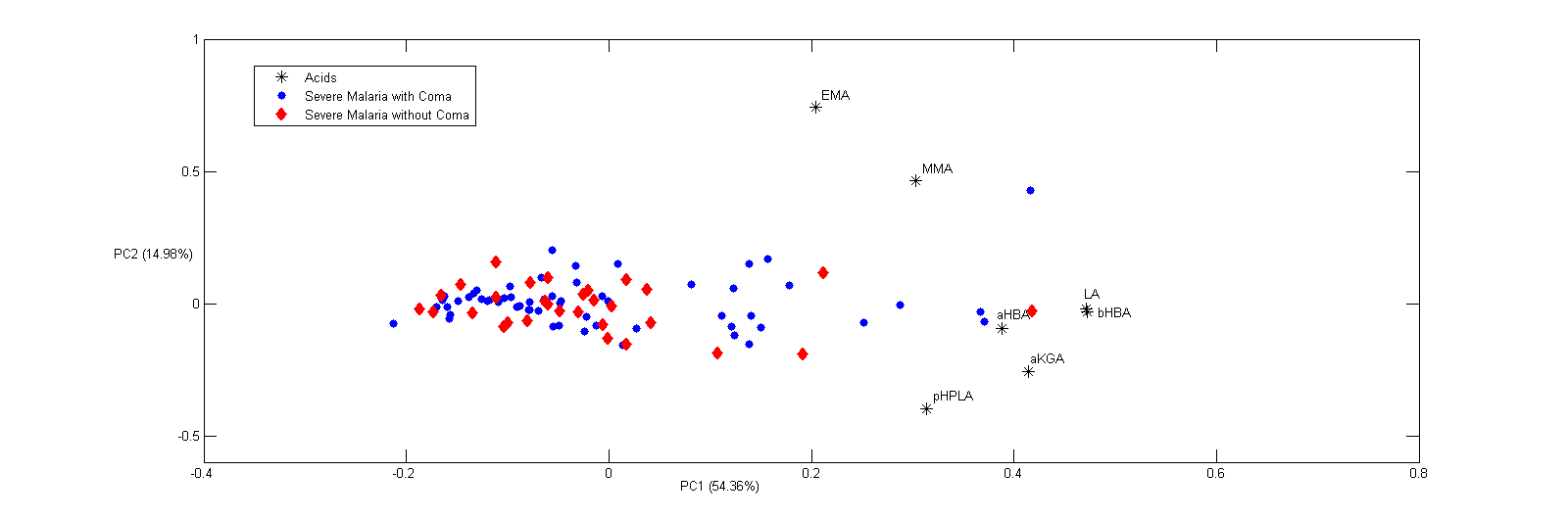


**Fig. S4** Principal Component Analysis (PCA) results of corrected urine concentrations of L-lactic acid (LA), α-hydroxybutyric acid (αHBA), β-hydroxybutyric acid (βHBA), p-hydroxyphenyllactic acid (HPLA), methylmalonic acid (MMA), ethylmalonic acid (EMA) and α-ketoglutaric acid (αKGA) of severe malaria patients with coma (in blue) and without coma (in red).


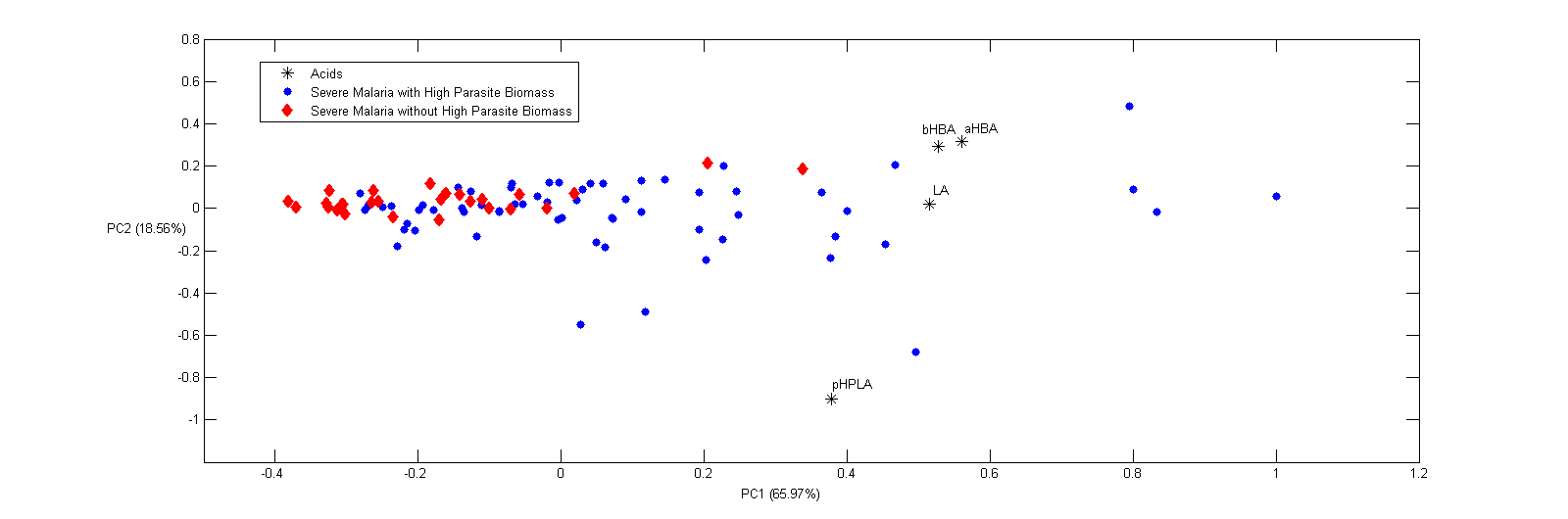


**Fig. S5** Principal Component Analysis (PCA) results of plasma concentrations of L-lactic acid (LA), α-hydroxybutyric acid (αHBA), β-hydroxybutyric acid (βHBA) and p-hydroxyphenyllactic acid (HPLA) of severe malaria patients with high parasite biomass (in blue) and without high parasite biomass (in red).


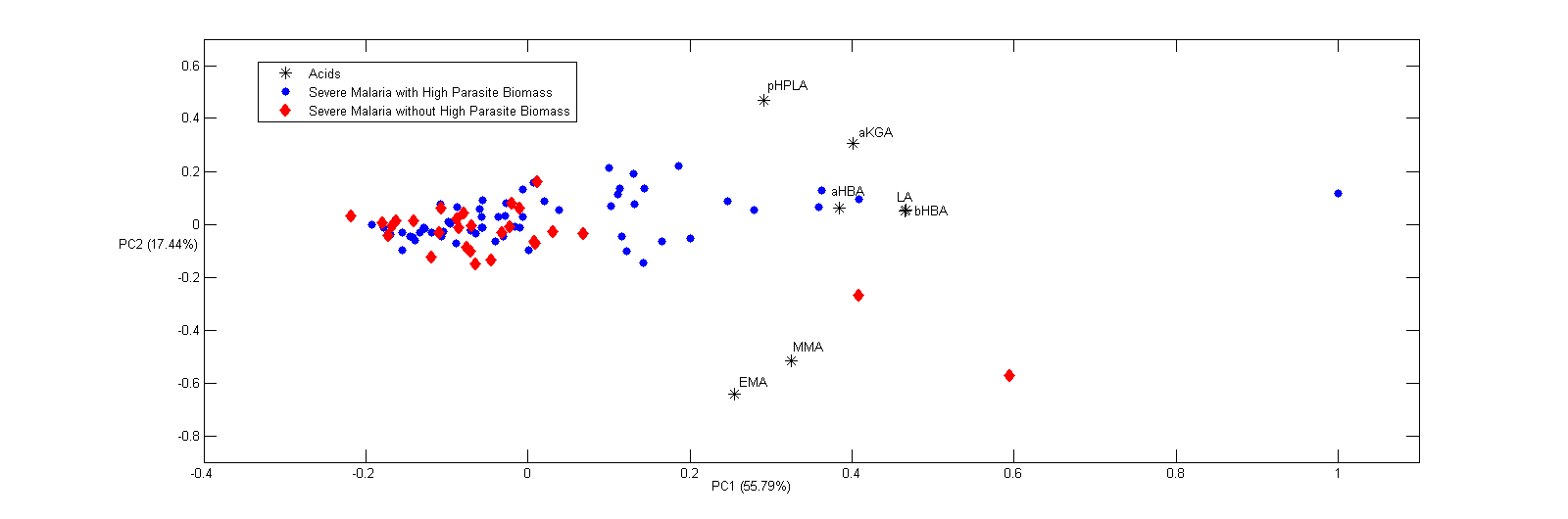


**Fig. S6** Principal Component Analysis (PCA) results of corrected urine concentrations of L-lactic acid (LA), α-hydroxybutyric acid (αHBA), β-hydroxybutyric acid (βHBA), p-hydroxyphenyllactic acid (HPLA), methylmalonic acid (MMA), ethylmalonic acid (EMA) and α-ketoglutaric acid (αKGA) of severe malaria patients with high parasite biomass (in blue) and without high parasite biomass (in red).

**
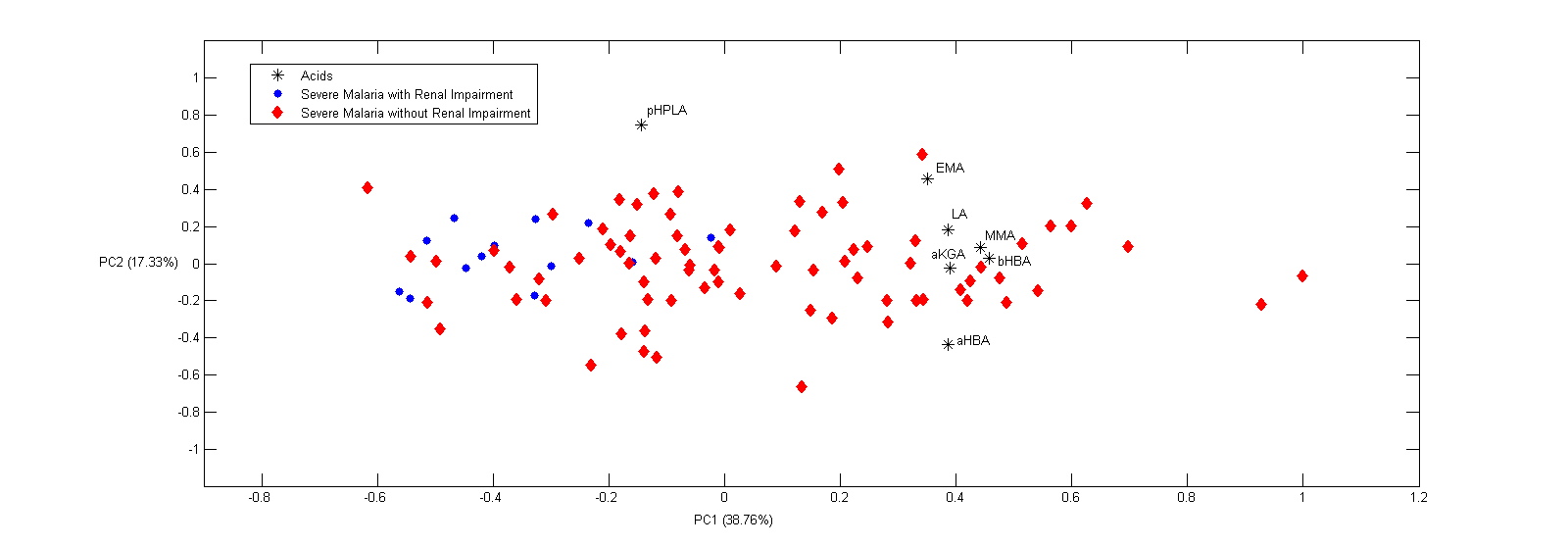
**

**Fig. S7** Principal Component Analysis (PCA) results of uncorrected urine concentrations of L-lactic acid (LA),

α-hydroxybutyric acid (αHBA), β-hydroxybutyric acid (βHBA), p-hydroxyphenyllactic acid (HPLA), methylmalonic acid (MMA), ethylmalonic acid (EMA) and α-ketoglutaric acid (αKGA) of severe malaria patients with AKI (in blue) and without AKI (in red**).**


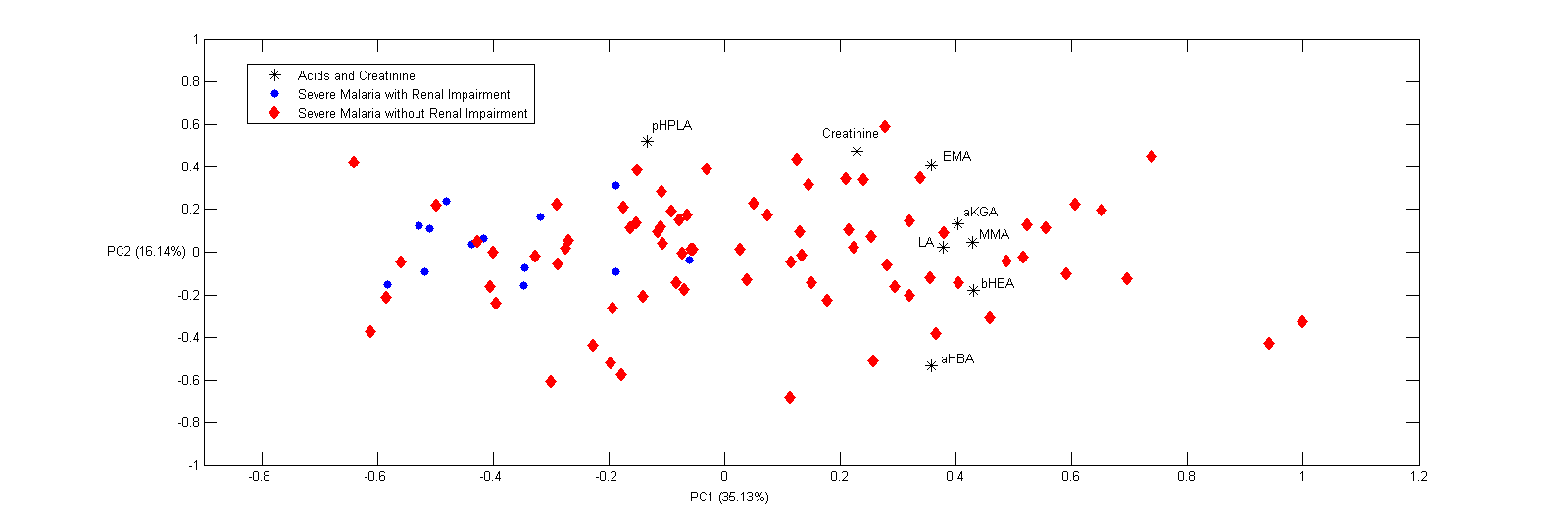


**Fig. S8** Principal Component Analysis (PCA) results of uncorrected urine concentrations of L-lactic acid (LA),

α-hydroxybutyric acid (αHBA), β-hydroxybutyric acid (βHBA), p-hydroxyphenyllactic acid (HPLA), methylmalonic acid (MMA), ethylmalonic acid (EMA) and α-ketoglutaric acid (αKGA) with urinary creatinine of severe malaria patients

with AKI (in blue) and without AKI (in red).

**Table S1a**

Plasma and urine concentration range of organic acids detected in patients with severe malaria with coma (+/-)

| Characteristics | Severe malaria | Severe malaria | P value |
| --- | --- | --- | --- |
|  | with coma (N=59) | without coma (N=31) |  |
| Plasma concentration (μ mol/L)^a^ |  |  |  |
| LA | 5177 (4272-6274) | 4077 (3308-5025) | 0.071 |
| αHBA | 130 (111-152) | 96.1 (76.1-121) | 0.083 |
| βHBA | 155 (133-182) | 133 (102-173) | 0.726 |
| pHPLA | 10.6 (9.20-12.2) | 9.70 (7.60-12.3) | 0.905 |
| Corrected urine concentrations |  |  |  |
| (μ mol/mmol CrCl)^a,b^ |  |  |  |
| LA | 11.0 (7.30-16.6) | 12.9 (7.40-22.4) | 0.333 |
| αHBA | 1.05 (0.73-1.51) | 0.98 (0.57-1.70) | 0.350 |
| βHBA | 2.71 (2.11-3.48) | 3.02 (2.01-4.56) | 0.232 |
| pHPLA | 1.51 (1.06-2.15) | 1.84 (1.18-2.88) | 0.758 |
| MMA | 0.15 (0.12-0.19) | 0.13 (0.09-0.18) | 0.536 |
| EMA | 0.22 (0.17-0.28) | 0.20 (0.15-0.27) | 0.477 |
| αKGA | 2.59 (2.07-3.24) | 2.90 (2.06-4.08) | 0.382 |

**Table S1b**

Plasma and urine concentration range of organic acids detected in patients with severe malaria with high biomass (+/-)

| Characteristics | Severe malaria | Severe malaria | P value |
| --- | --- | --- | --- |
|  | with high parasite biomass (N=62) | without high parasite biomass (N=28) |  |
| Plasma concentration (μ mol/L)^a^ |  |  |  |
| LA | 5774 (4888-6821) | 3194 (2527-4037) | 0.001 |
| αHBA | 139 (120-161) | 82.9 (66.2-104) | 0.005 |
| βHBA | 168 (142-198) | 107 (89.9-127) | 0.048 |
| pHPLA | 12.5 (10.7-14.6) | 7.03 (6.41-7.72) | 0.002 |
| Corrected urine concentrations |  |  |  |
| (μ mol/mmol CrCl)^a,b^ |  |  |  |
| LA | 15.5 (10.6-22.7) | 7.30 (3.93-13.6) | 0.404 |
| αHBA | 1.21 (0.85-1.72) | 0.82 (0.45-1.50) | 0.363 |
| βHBA | 3.21 (2.44-4.23) | 2.31 (1.62-3.29) | 0.444 |
| pHPLA | 2.05 (1.47-2.84) | 0.99 (0.62-1.59) | 0.024 |
| MMA | 0.15 (0.12-0.19) | 0.14 (0.09-0.21) | 0.582 |
| EMA | 0.20 (0.17-0.24) | 0.27 (0.15-0.47) | 0.021 |
| αKGA | 2.91 (2.28-3.70) | 2.37 (1.80-3.12) | 0.094 |

Legend: values are geometric mean (95% CI)

^a^For all plasma specimens, MMA, EMA and αKGA were assayed but undetectable.

^b^Corrected urine concentrations were adjusted for impaired creatinine clearance by incorporating the urine/plasma creatinine ratio

**Table S2**

Summary of linear regression models in patients with severe falciparum malaria (N = 90), with plasma PfHRP2 concentrations and plasma or urinary acid concentrations as independent variables, and plasma or urinary creatinine concentrations as dependent variable

|  | Univariate analysis | |  | Multivariate analysis | |  |
| --- | --- | --- | --- | --- | --- | --- |
|  | β | (95 % CI) | P value* | β | (95 % CI) | P value* |
| Regression of plasma acid concentrations and plasma PfHRP2 | | | |  |  |  |
| LA | 0.224 | (0.021 - 0.428) | 0.031 | -0.138 | (-0.274 - -0.001) | 0.048 |
| αHBA | 0.187 | (-0.049 - 0.424) | 0.119 | - |  |  |
| βHBA | 0.363 | (0.093 - 0.633) | 0.009 | - |  |  |
| pHPLA | 0.770 | (0.648 - 0.892) | 0.000 | 0.827 | (0.694 - 0.960) | 0.000 |
| PfHRP2 | 0.174 | (0.081 - 0.269) | 0.000 | - |  |  |
| Regression of corrected urine acid concentrations and plasma PfHRP2 | | | | |  |  |
| LA | 0.183 | (0.889 - 0.277) | 0.000 | - |  |  |
| αHBA | 0.035 | (-0.070 - 0.139) | 0.510 | - |  |  |
| βHBA | 0.292 | (0.137 - 0.446) | 0.000 | -0.175 | (-0.330 - -0.020) | 0.027 |
| pHPLA | 0.325 | (0.246 - 0.405) | 0.000 | 0.226 | (0.135 - 0.317) | 0.000 |
| MMA | -0.101 | (-0.257 - 0.054) | 0.199 | - |  |  |
| EMA | -0.077 | (-0.225 - 0.071) | 0.307 | - |  |  |
| αKGA | 0.561 | (0.424 - 0.699) | 0.000 | 0.454 | (0.270 - 0.639) | 0.000 |
| PfHRP2 | 0.125 | (0.038 - 0.212) | 0.005 | - |  |  |

Abbreviations: L-lactic acid (LA), α-hydroxybutyric acid (αHBA), β-hydroxybutyric acid (βHBA), p-hydroxyphenyllactic acid (pHPLA), methylmalonic acid (MMA), ethylmalonic acid (EMA), α-ketoglutaric acid (αKGA) and Plasmodium falciparum histidine-rich protein 2 (PfHRP2)

**Table S3**

Summary of linear regression models in patients with sepsis (N = 19), with plasma PfHRP2 concentrations and plasma or urinary acid concentrations as independent variables, and plasma or urinary creatinine concentrations as dependent

|  | Univariate analysis | |  | Multivariate analysis | |  |
| --- | --- | --- | --- | --- | --- | --- |
|  | β | (95 % CI) | P value* | β | (95 % CI) | P value |
| Regression of plasma acid concentrations and plasma PfHRP2 | | | |  |  |  |
| LA | 0.133 | (-0.383 - 0.650) | 0.599 | - |  |  |
| αHBA | -0.005 | (-0.589 - 0.579) | 0.986 | - |  |  |
| βHBA | 0.062 | (-0.251 - 0.374) | 0.687 | - |  |  |
| pHPLA | 0.849 | (0.253 - 1.444) | 0.007 | - |  |  |
| Regression of corrected urine acid concentrations and plasma PfHRP2 | | | | |  |  |
| LA | -0.015 | (-0.219 - 0.190) | 0.879 | - |  |  |
| αHBA | -0.041 | (-0.368 - 0.285) | 0.793 | - |  |  |
| βHBA | -0.054 | (-0.336 - 0.228) | 0.690 | - |  |  |
| pHPLA | 0.138 | (-0.0490 - 0.325) | 0.140 | - |  |  |
| MMA | 0.366 | (-0.147 - 0.879) | 0.150 | - |  |  |
| EMA | 0.281 | (-0.131 - 0.694) | 0.168 | - |  |  |
| αKGA | 0.217 | (-0.232 - 0.665) | 0.322 | - |  |  |

**Abbreviations:** L-lactic acid (LA), α-hydroxybutyric acid (αHBA), β-hydroxybutyric acid (βHBA), p-hydroxyphenyllactic acid (HPLA), methylmalonic acid (MMA), ethylmalonic acid (EMA) and α-ketoglutaric acid (αKGA)

**Table S4a**

Partial Least Square Discriminant Analysis classification results of plasma concentration of 4 acids

|  |  | Predicted sample groups | | |
| --- | --- | --- | --- | --- |
|  |  | Severe malaria with AKI | Severe malaria without AKI | Correct classification (%) |
| Original data set | Severe malaria with AKI | 11 | 2 | 84.6 |
|  | Severe malaria without AKI | 0 | 77 | 100 |
|  | Recognition ability (%) |  |  | 92.3 |
| Cross-validation | Severe malaria with AKI | 11 | 2 | 84.6 |
|  | Severe malaria without AKI | 0 | 77 | 100 |
|  | Predictive ability (%) |  |  | 92.3 |

**Table S4b**

Partial Least Square Discriminant Analysis classification results of corrected urine concentration of 7 acids

|  | Predicted sample groups | | | |
| --- | --- | --- | --- | --- |
|  |  | Severe malaria with AKI | Severe malaria without AKI | Correct classification (%) |
| Original data set | Severe malaria with AKI | 13 | 0 | 100 |
|  | Severe malaria without AKI | 1 | 76 | 98.7 |
|  | Recognition ability (%) |  |  | 99.4 |
| Cross-validation | Severe malaria with AKI | 13 | 0 | 100 |
|  | Severe malaria without AKI | 1 | 76 | 98.7 |
|  | Predictive ability (%) |  |  | 99.4 |
